# Pancreatic Stenting Reduces Post-ERCP Pancreatitis and Biliary Sepsis in High-Risk Patients: A Randomized, Controlled Study

**DOI:** 10.1155/2016/9687052

**Published:** 2016-02-29

**Authors:** He-Kun Yin, Hai-En Wu, Qi-Xiang Li, Wei Wang, Wei-Lin Ou, Harry Hua-Xiang Xia

**Affiliations:** ^1^Department of Gastroenterology, Jiangmen Central Hospital, Jiangmen, Guangdong, China; ^2^Department of Gastroenterology, The First Hospital Affiliated to Guangdong Pharmaceutical University, Guangzhou, Guangdong, China

## Abstract

*Background*. Endoscopic retrograde cholangiopancreatography (ERCP) is an established treatment modality for bile duct disorders, but patients have a risk of post-ERCP pancreatitis (PEP) and biliary sepsis.* Aim*. To evaluate the effectiveness and safety of pancreatic stent for prophylaxis of PEP and biliary sepsis in high-risk patients with complicating common bile duct (CBD) disorders.* Methods*. Two hundred and six patients with complicating confirmed or suspected CBD disorders were randomly assigned to receive ERCP with pancreatic stenting (experimental group) or without stenting (control group). Primary outcome measure was frequency of PEP, and secondary outcome measures included operative time, blood loss, postoperative recovery times, and other ERCP-associated morbidities.* Results*. Baseline age, sex, CBD etiology, concomitant medical/surgical conditions, cannulation difficulty, and ERCP success were comparable between the two groups (all *P* > 0.05). Compared to the control group, the experimental group had a significantly lower frequency of PEP (7.7%* versus* 17.7%, *P* < 0.05) and positive bile microbial culture (40.4%* versus* 62.7%, *P* < 0.05). However, the two groups were similar in operative time, blood loss, postoperative recovery times, and other ERCP-associated morbidities (all *P* > 0.05).* Conclusions*. Pancreatic stenting can reduce the occurrence of PEP and biliary sepsis in high-risk patients with complicating CBD disorders but does not increase other ERCP-associated morbidities. This trial is registered with the Chinese Clinical Trial Registry (registration identifier ChiCTR-OCH-14005134).

## 1. Introduction

Endoscopic retrograde cholangiopancreatography (ERCP) is an advanced endoscopic technique for the diagnosis and treatment of bile and pancreatic duct disorders, such as gallstones, inflammatory stricture, and cancer [1]. Combined with other noninvasive diagnostic techniques such as endoscopic ultrasonography [2] and magnetic resonance cholangiopancreatography [3], ERCP represents an accurate diagnostic alternative. Moreover, ERCP with interventional endoscopic techniques, such as endoscopic sphincterotomy [4] and stenting [5], offers an effective and safe option for treatment of surgically indicated bile and pancreatic duct disorders. Generally speaking, therapeutic ERCP is generally safe and associated with an expedited postoperative recovery; however, patients are at a certain risk for ERCP-associated morbidities, such as post-ERCP pancreatitis (PEP), gastrointestinal bleeding/perforation, contrast medium anaphylaxis, cardiopulmonary insufficiency, bile/pancreatic duct infection (septic cholangitis or pancreatitis), and even mortality in rare cases [6]. These ERCP-associated complications will impair patients' general well-being and quality of life and increase the public healthcare burden.

PEP is the most common and a serious complication of ERCP, with a reported 2−10% incidence rate among unselected patients, 2–4% among low-risk patients, and even up to 8–40% among high-risk patients [7–13]. PEP is usually mild in severity and self-limiting in duration but often requires medical and even surgical intervention, especially in patients with related risk factors. Risk factors contributing to PEP include young age, female gender, previous history of cholangitis or pancreatitis, prior post-ERCP pancreatitis, normal serum bilirubin, recurrent pancreatitis, sphincter of Oddi dysfunction, repeated bile/pancreatic duct accesses, iatrogenic procedural injury, presence of gallstones, periampullary duodenal diverticulum, and insufficient pancreatic drainage [13–17]. Medical intervention, such as use of somatostatin [18], gabexate mesilate [19], nitroglycerin [20], antimicrobial agent, and nonsteroidal anti-inflammation agent [21], has been attempted but exhibits a controversial prophylactic role among previous reports except for non-steroidal anti-inflammation agent. Endoscopic intervention, such as placement of a nasal biliary drainage tube, is reported to be effective for preventing cholestasis and cholangitis but offers a limited prophylactic effect in cases of insufficient pancreatic duct drainage.

Placement of a pancreatic stent has been reported to be an effective, safe prophylactic, and therapeutic regimen for multiple pancreatic pathologies, such as pancreatitis including PEP [22], pancreatic gallstones [23], traumatic injury [24], fistula [25], and stricture [26] with respect to both occurrence and severity. The primary objective of this study was to evaluate whether pancreatic stenting could reduce the occurrence of PEP and biliary sepsis in high-risk patients with complicating common bile duct (CBD) disorders, the most frequent indication for ERCP among Eastern Asian populations, in an assessor-blinded, randomized, controlled study setting.

## 2. Patients and Methods

### 2.1. Study Protocol

The study protocol was approved by the Institutional Review Board at Jiangmen Central Hospital in accordance with the latest version of the Declaration of Helsinki and registered with the Chinese Clinical Trial Registry (http://www.chictr.org.cn/; registration identifier ChiCTR-OCH-14005134). Two hundred and six patients with confirmed or suspected benign or malignant CBD disorders were hospitalized at our Department of Gastroenterology for elective ERCP between December 2009 and May 2014. The inclusion criteria were as follows: age greater than 18 years; presence of clinically significant abdominal pain, nausea/vomiting, or jaundice; confirmed benign or malignant bile duct disorders diagnosed on ultrasonography, computed tomography scan, or magnetic resonance cholangiopancreatography; normal baseline serum amylase level; and being indicated for therapeutic ERCP due to benign and malignant bile and pancreatic duct disorders [27, 28]. Risk factors for PEP included age less than 60 years, female sex, previous history of bile/pancreatic duct surgery/endoscopy, prior post-ERCP pancreatitis, cannulation difficulty, complicating gallstones or periampullary duodenal diverticulum, and normal serum bilirubin level [13–17, 27–31]. All patients with at least two risk factors for PEP were included. The exclusion criteria were as follows: pregnant or lactating; allergic to nonionic contrast medium; presence of complicating acute pancreatitis or active chronic pancreatitis, or choledochoduodenostomy; complicating small bowel stricture or obstruction; complicating serious cerebrovascular, cardiopulmonary, or hepatorenal impairment; complicating psychological or psychiatric conditions; or rejection to participate in this study. All patients volunteered to give informed consenting prior to participation in this study.

### 2.2. ERCP Procedure

All patients provided samples for hematologic, clinical biochemistry, serologic, and virologic assays, including those for serum lipid, amylase, and lipase, as well as ultrasonography, computed tomography scanning, and magnetic resonance cholangiopancreatography. Patients were instructed to fast 8 hours prior to ERCP and received a contrast medium skin allergy test. Premedications included intramuscular 10-mg anisodamine, 10-mg diazepam, and 50-mg pethidine.

All ERCP procedures were performed by an assigned endoscopic team led by a board-certified endoscopic gastroenterologist (the corresponding author). All eligible patients were randomly and equally assigned to receive either ECRP with pancreatic stenting (experimental group) or without stenting (control group) by using a random number table. ERCP was performed as routine; briefly, the duodenal papilla was identified using a duodenal endoscope (FUJINON-530; Fujifilm, Tokyo, Japan) followed by sequential insertion of the guidewire, cholangiopancreatography, ampullary sphincterotomy, balloon dilation or gallstone basketing, and placement of a nasal biliary drain. In the experimental group, an additional 5, 7, or 9 cm long 5 Fr plastic stent (Endo-Flex GmbH, Voerde, Germany) was placed after ampullary sphincterotomy and pancreatic duct contrast radiography.

Cholangiopancreatography was done before pancreatic stenting to determine the location and length of CBD disease, as well as the opening, length, and dilation of the bile and pancreatic ducts. Generally a 5 cm 5 Fr stent was applied if the pancreatic duct was less than 2 mm in diameter, and a 7 or 9 cm stent was used when the pancreatic duct diameter was more than 2 mm.

### 2.3. Post-ERCP Care

Patients were instructed to fast for 72 hours after ERCP and given continuous intravenous infusion of prophylactic antimicrobial agent and somatostatin for 12 successive hours. Serial follow-up assays included routine hematologic, liver function, serum lipid, amylase, lipase, and bile biochemical tests as well as bile microbiologic culture. Pancreatic stents were removed using endoscopy at least 72 hours after ERCP if the serum amylase level remained within the normal limit, the stent showed good positioning on plain abdominal radiography, and no residual gallstones were detectable on an abdominal computed tomography scan. Repeated ERCP was performed to remove any residual gallstones.

### 2.4. Definitions and Outcome Measures

PEP was defined as the emergence of any symptoms suggestive of pancreatitis, such as newly onset or worsening abdominal pain, persisting for more than 24 hours and a serum amylase level more than 3 times the upper limit of normal; PEP resolution was defined as the disappearance of any symptom suggestive of pancreatitis and return of serum amylase to within the normal limit [23]. ERCP success referred to successful contrast radiography of the bile and pancreatic ducts. Cannulation difficulty was defined as a bile duct cannulation completed over 10 minutes or more than five attempts due to mistaken access to the pancreatic duct.

The primary outcome measure was frequency of PEP, and secondary outcome measures included operative time, blood loss, postoperative recovery times, and other ERCP-associated morbidities.

### 2.5. Statistical Analysis

The SPSS 17.0 statistical software package (SPSS Inc., Chicago, IL, USA) was used for statistical analysis. All continuous data are expressed as mean ± standard deviation, and the means were compared using the two independent samples Student *t*-test or one-way or repeated measures analysis of variance. All categorical data were expressed as *n* (%) and compared using the Fisher exact probability or log-rank test. A *P* value less than 0.05 was considered statistically significant.

## 3. Results

### 3.1. Baseline Patient Characteristics

**Figure 1:**
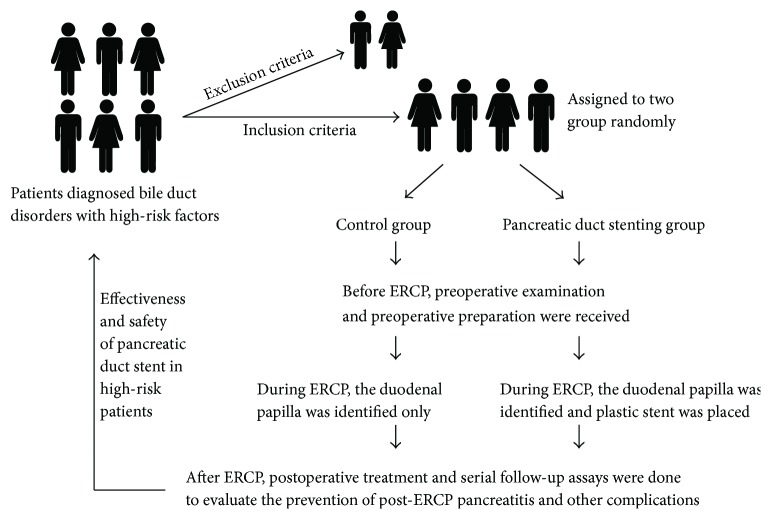
Patient assignment flowchart.

Overall 206 patients, including 114 men and 92 women with a mean age of 59 years (range, 21–88 years), were eligible for inclusion in this study (Figure 1). The baseline patient characteristics are shown in Table 1. Underlying biliary and pancreatic disorders included common bile duct gallstones (*n* = 132), bile duct dilation of unknown cause (*n* = 8), cholangitis (*n* = 3), malignant common bile duct stricture (*n* = 45), pancreatic cancer (*n* = 13), duodenal papillitis (*n* = 3), and sclerosing cholangitis (*n* = 2). Notably, 63 of 206 (30.6%) patients had concomitant clonorchiasis due to an endemic prevalence. The two groups were comparable in terms of age, sex, body mass index, biliary/pancreatic disorders, and concomitant medical/surgical conditions (all *P* > 0.05).

### 3.2. Operative Data

The operative data are shown in Table 2. The two groups had a similar overall operative time (*P* > 0.05). ERCP was uneventfully completed in 199 of 206 (96.6%) patients, whereas 181 of 206 (87.9%) patients presented difficulty in cannulation. Concomitant periampullary diverticulum was identified in 17 of 206 (8.3%) patients. These three measures were similar between the two groups (all *P* > 0.05).

### 3.3. PEP and Other ERCP-Associated Complications

**Figure 2:**
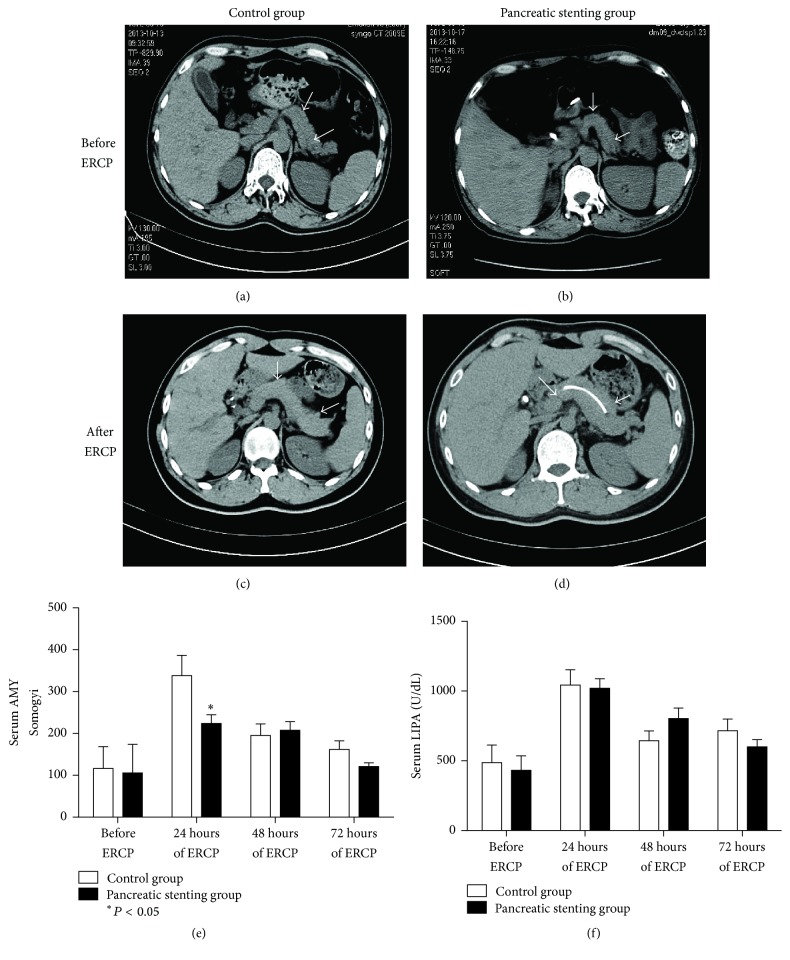
Occurrence and resolution of PEP: (a–d) representative computed tomography scan of the experimental (with pancreatic duct stenting) and control groups (without stenting) before and after ERCP showing obvious PEP (as indicated by the white arrow) in the control group; serum levels of (e) amylase and (f) lipase before ERCP and 24 h, 48 h, and 72 h after ERCP.

PEP and other ERCP-associated complications are shown in Table 3. Overall, PEP occurred in 26 of 206 (12.6%) patients. The experimental group had a significantly lower frequency of PEP (Figures 2(a)–2(d)) than the control group (experimental versus control, 7.7% [8/104] versus 17.7% [18/102], *P* < 0.05). Compared to those before ERCP, the two groups exhibited a significant increase in serum levels of amylase (Figure 2(e)) and lipase (Figure 2(f)) at 24 h after ERCP (both *P* < 0.05). These changes were significantly less extent in the experimental group (*P* < 0.05) and remained similar at 48 h and 72 h after ERCP (both *P* > 0.05). However, PEP resolved in both groups after medical treatment within a similar time frame (3.0 ± 1.2 d versus 3.1 ± 2.0 d, *P* > 0.05). Other ERCP-associated morbidities included postoperative bleeding resolution after use of a hemostatic (*n* = 1, 0.5%) and requirement of second-look ERCP due to residual gallstones (*n* = 1, 0.5%). Pancreatic stent displacement occurred in 4 of 104 (3.9%) patients in the experimental group, and the displaced stent was removed using endoscopy (*n* = 3) or left untreated (*n* = 1) without clinically significant sequelae. None of patients required repeated ERCP due to PEP, and no mortality occurred.

### 3.4. Follow-Up Laboratory Data

**Figure 3:**
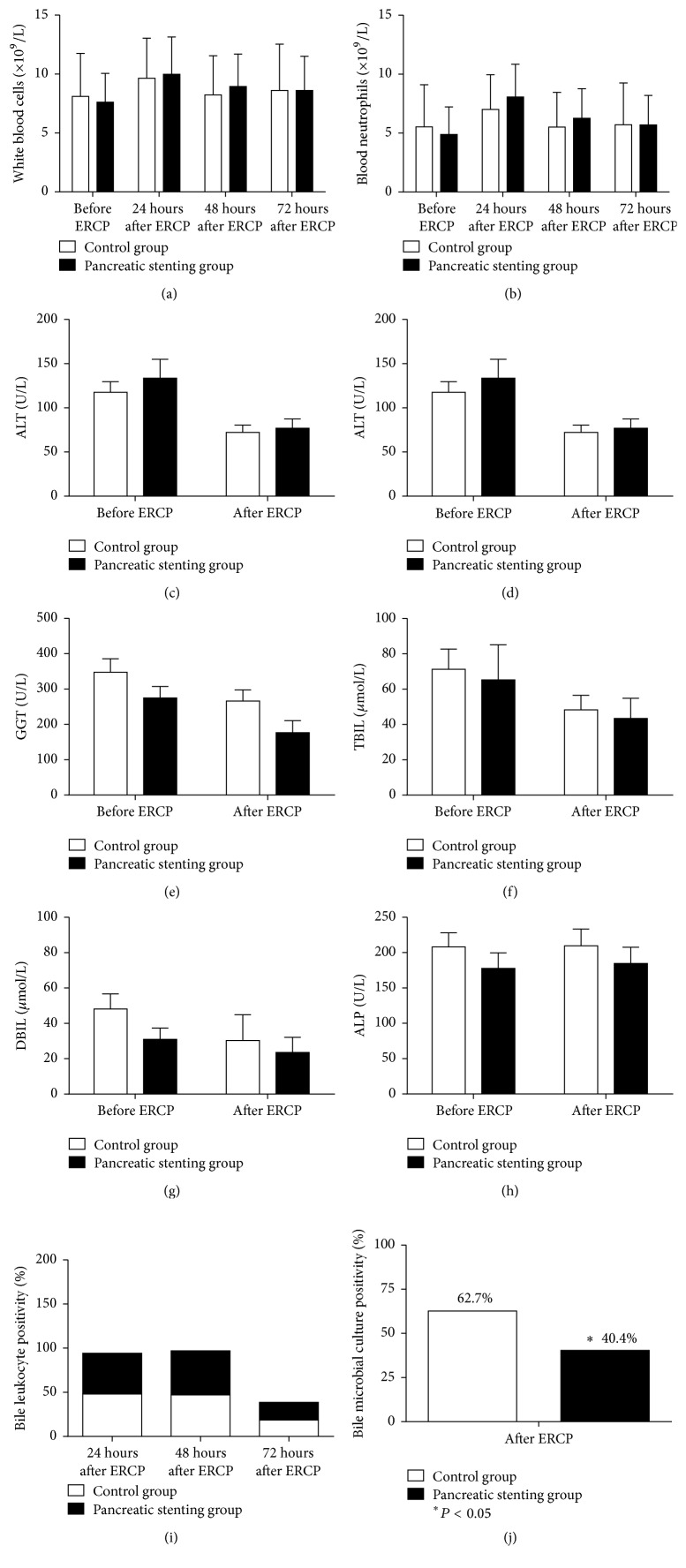
Follow-up laboratory data: blood (a) leukocyte and (b) neutrophil counts; serum levels of (c) alanine aminotransferase, (d) aspartate aminotransferase, (e) gamma-glutamyl transpeptidase, (f) total bilirubin, (g) direct bilirubin, (h) alkaline phosphatase, (i) bile leukocyte positivity, and (j) bile microbial culture.

Compared to baseline counts, both groups exhibited a transient increase in blood leukocyte and neutrophil counts with no significant differences before ERCP and at 24 h, 48 h, and 72 h after ERCP (*P* > 0.05; Figures 3(a) and 3(b)). Moreover, both groups experienced similar and significant reductions (all *P* > 0.05) in serum levels of alanine aminotransferase (Figure 3(c)), aspartate aminotransferase (Figure 3(d)), gamma-glutamyl transpeptidase (Figure 3(e)), total bilirubin (Figure 3(f)), and direct bilirubin (Figure 3(g)) compared to baseline levels. However, the alkaline phosphatase level remained unchanged in the two groups before and after ERCP (both *P* > 0.05; Figure 3(h)).

Positivity for bile leukocytes was observed in 97 of 206 (47.1%), 100 of 206 (48.5%), and 40 of 206 (19.4%) patients at 0, 24, and 48 h after ERCP; the two groups had a similar positivity for bile leukocytes (all *P* > 0.05; Figure 3(i)). However, the experimental group showed that a significantly lower percentage of these patients have a positive bile microbial culture compared to patients in the control group (42/104 [40.4%]* versus* 64/102 [62.7], *P* < 0.05; Figure 3(j)). Major pathogenic microbes included* Escherichia coli* (*n* = 13, 6.3%),* E. faecalis *(*n* = 21, 10.2%), and* C. albicans* (*n* = 22, 10.7%), and all these infections resolved after sensitive antimicrobial treatment.

## 4. Discussion

Elevation in serum amylase occurs in as many as 75% of patients after ERCP [11, 32] and reaches a peak at 24 hours after ERCP as shown by our results, whereas PEP, namely, acute clinical pancreatitis manifesting as abdominal pain and hyperamylasemia occurs in a relatively small portion of patients but varies among reports [13]. Haciahmetoglu et al. [33] proposed that this variation might result from differences in the definition of PEP, data collection method, and, especially, inclusion of patients with preexisting risk factors or not. The overall frequency of PEP was 12.6% in our patients, who had at least two risk factors, similar to those at a higher risk reported by Sofuni et al. [34]. It was noted that our patients were prospectively found to be at a relatively higher risk while the high-risk patient cohort reported by Sofuni et al. [34] identified. The fundamental pathogenesis underlying PEP is mechanical injury from endoscopic instrumentation [35] and hydrostatic injury from contrast medium injection [36] on the pancreatic duct. Independent and dependent predisposing factors for PEP are categorized into patient- and procedure-related factors [36]: the former category mainly includes age below 60 years, female, previous history of acute or chronic pancreatitis, and normal serum bilirubin level, and the latter ones primarily include ampullary manipulation, repeated cannulation, use of the precut technique, and operator's experience.

A major pathophysiological mechanism underlying PEP is insufficient pancreatic duct drain and/or increased pancreatic duct hydrostatic pressure after ERCP. Some retrospective studies and meta-analyses demonstrated that prophylactic placement of pancreatic stent could significantly reduce the risk of PEP in high-risk patients by approximately 70%–80% [37, 38]. Previous studies also suggested that use of a larger-caliber stent and a polyethylene stent was associated with a significantly lower risk of PEP than the use of a smaller-caliber stent and of metallic stent, respectively [39, 40]. As displacement and removal of a retained pancreatic duct confer a risk for PEP, current consensus regarding prophylactic pancreatic stenting after ERCP recommends that stenting should only be given in high-risk patients, such as those with iatrogenic ampullary injury, inadvertent pancreatic duct injection, and residual gallstones [41]. Our results showed that prophylactic use of a pancreatic stent could significantly reduce the occurrence of PEP from 17.7% to 7.7% in high-risk patients in a randomized controlled study setting. However, the two groups were similar in times of PEP resolution with respect to clinical symptoms and serum amylase level as well as other ERCP-associated morbidities. This finding suggested that pancreatic stenting has a short- rather than long-term effect and a prophylactic rather than a therapeutic effect on PEP, necessitating the requirement of early stenting in patients with preexisting risk factors.

Pathophysiologically PEP is an iatrogenic acute pancreatitis secondary to ERCP elicited by a series of locoregional and/or systemic inflammatory cascade reactions. Our results showed that blood leukocyte and neutrophil counts exhibited a similar transient increase in both groups, suggesting a nonspecific systemic inflammatory response to ERCP rather than pancreatic stenting. Moreover, the similar improvement in liver function measures, especially those indicative of biliary tract drain sufficiency, between the two groups indicated that additional placement of pancreatic stent had no adverse effect on post-ERCP bile drain. A possible extra benefit of pancreatic stenting was reduction in potential risk of biliary sepsis as shown by the lower percentage of bile microbial culture positivity in the experimental group, although the two groups were comparable in bile leukocyte positivity. A possible explanation is that sufficient pancreatic duct drainage also helps to improve bile duct drainage as the two ductal systems share a common opening to the duodenum. Placement of the pancreatic stent led to sufficient drainage of pancreatic juice through the pancreatic duct to the duodenum, which could reduce the digestive effect of pancreatic enzymes and bacterial colonization through the duodenal papilla. Combined with the inhibitive effect of somatostatin on the Oddi's sphincter, pancreatic stenting could restore the Oddi's sphincter and pancreatic duct drainage [42, 43], which would further inhibit bacterial colonization and invasion.

There were some limitations in this study. First, this study was not investigator-blinded due to the requirement of pancreatic stenting. However, all ERCP procedures were performed by a single endoscopic team in a randomized setting. Secondly, PEP occurrence was not stratified by the severity of pancreatitis, which might underestimate the therapeutic effect of stenting on PEP with respect to PEP resolution time. However, previous reports suggested that ERCP itself was associated with the odds rather than severity of PEP [36]. Lastly, our results demonstrate the mid- or long-term efficacy and safety data after ERCP with or without pancreatic stenting as the primary study objective focused on the prophylactic effect of pancreatic stenting in PEP.

In conclusion, PEP is a common morbidity after ERCP, especially in high-risk patients with complicating CBD disorders. However, use of pancreatic stenting can significantly reduce the PEP risk in these patients by improving pancreatic duct drainage, although it does not expedite recovery from PEP. The presence of a pancreatic stent has a beneficial rather than adverse effect on bile duct drain. Long-term follow-up studies are required to validate the long-term efficacy and safety of additional pancreatic stenting for high-risk patients with complicating CBD disorders regarding PEP and other ERCP-associated morbidities.

## Figures and Tables

**Table 1:** Baseline demographic and clinical characteristics of patients.

	Experimental group (*n* = 104)	Control group (*n* = 102)	*P*
Age, year, mean ± SD	57.2 ± 14.4	57.4 ± 13.9	0.924
Sex, male/female	59/45	55/47	0.685
ERCP indications, *n* (%)			
CBD gallstone	72 (69.2)	60 (58.8)	0.120
Bile duct dilation	3 (2.9)	5 (4.9)	0.454
Cholangitis	2 (1.9)	1 (1.0)	0.572
Malignant CBD stricture	24 (23.1)	34 (33.3)	0.102
Pancreatic cancer	9 (8.7)	4 (3.9)	0.163
Duodenal papillitis	2 (1.9)	1 (1.0)	0.572
Sclerosing cholangitis	1 (1.0)	1 (1.0)	0.989
Concomitant liver fluke disease, *n* (%)	31 (29.8)	32 (31.4)	0.807
Complicating risk factors, *n* (%)			
Complicating 2 risk factors	22 (21.2)	29 (28.4)	0.226
Complicating 3 risk factors	25 (24.0)	32 (31.4)	0.239
Complicating 4 risk factors	38 (36.5)	25 (24.5)	0.061
Complicating ≥ 5 risk factors	19 (18.3)	16 (15.7)	0.622

**Table 2:** Operative data of ERCP.

	Experimental group (*n* = 104)	Control group (*n* = 102)	*P*
Overall OT, min, mean ± SD	43 ± 14	40 ± 15	0.772
ERCP success, *n* (%)	101 (97.1)	98 (96.1)	0.681
Pancreatogram	101 (97.1)	98 (96.1)	0.681
Sphincterotomy	102 (98.1)	102 (100.0)	0.159
Cannulation difficulty, *n* (%)	93 (89.4)	88 (86.3)	0.489
Periampullary diverticulum, *n* (%)	9 (8.7)	8 (7.8)	0.833

**Table 3:** PEP and other ERCP-associated morbidities.

	Experimental group (*n* = 104)	Control group (*n* = 102)	*P*
PEP, *n* (%)	8 (7.7)	18 (17.7)	**0.031**
PEP recovery time, d, mean ± SD	3.0 ± 1.2	3.1 ± 2.0	0.829
Time to resume oral intake, d, mean ± SD	3.2 ± 1.8	3.5 ± 1.6	0.765
Postoperative hospital stay, d, mean ± SD	8.8 ± 3.5	8.5 ± 4.1	0.552
Postoperative bleeding, *n* (%)	0 (0.0)	1 (1.0)	0.311
Postoperative perforation, *n* (%)	0 (0.0)	0 (0.0)	N/A
Postoperative infection, *n* (%)	0 (0.0)	0 (0.0)	N/A
Second-look ERCP	1 (1.0)	0 (0.0)	0.321
PDS displacement, *n* (%)	4 (3.9)	N/A	N/A
Mortality, *n* (%)	0 (0.0)	0 (0.0)	N/A

N/A: not applicable.

## References

[1] Moon J. H., Choi H. J., Lee Y. N. (2014). Endoscopic retrograde cholangiopancreatography. *Endoscopy*.

[2] Singla V., Garg P. K. (2013). Role of diagnostic and therapeutic endoscopic ultrasonography in benign pancreatic diseases. *Endoscopic Ultrasound*.

[3] Wang C.-L., Ding H.-Y., Dai Y. (2014). Magnetic resonance cholangiopancreatography study of pancreaticobiliary maljunction and pancreaticobiliary diseases. *World Journal of Gastroenterology*.

[4] Cotton P. B., Durkalski V., Romagnuolo J. (2014). Effect of endoscopic sphincterotomy for suspected sphincter of oddi dysfunction on pain-related disability following cholecystectomy: the EPISOD randomized clinical trial. *The Journal of the American Medical Association*.

[5] Barkay O., Mosler P., Schmitt C. M. (2013). Effect of endoscopic stenting of malignant bile duct obstruction on quality of life. *Journal of Clinical Gastroenterology*.

[6] Glomsaker T., Hoff G., Kvaløy J. T., Søreide K., Aabakken L., Søreide J. A. (2013). Patterns and predictive factors of complications after endoscopic retrograde cholangiopancreatography. *British Journal of Surgery*.

[7] Lee T. H., Jung Y. K., Park S.-H. (2014). Preparation of high-risk patients and the choice of guidewire for a successful endoscopic retrograde cholangiopancreatography procedure. *Clinical Endoscopy*.

[8] Shi Q.-Q., Ning X.-Y., Zhan L.-L., Tang G.-D., Lv X.-P. (2014). Placement of prophylactic pancreatic stents to prevent post-endoscopic retrograde cholangiopancreatography pancreatitis in high-risk patients: a meta-analysis. *World Journal of Gastroenterology*.

[9] Navaneethan U., Konjeti R., Venkatesh P. G., Sanaka M. R., Parsi M. A. (2014). Early precut sphincterotomy and the risk of endoscopic retrograde cholangiopancreatography related complications: an updated meta-analysis. *World Journal of Gastrointestinal Endoscopy*.

[10] Dimagno M. J., Spaete J. P., Ballard D. D., Wamsteker E.-J., Saini S. D. (2013). Risk models for post-endoscopic retrograde cholangiopancreatography pancreatitis (PEP): smoking and chronic liver disease are predictors of protection against PEP. *Pancreas*.

[11] Freeman M. L., Guda N. M. (2004). Prevention of post-ERCP pancreatitis: a comprehensive review. *Gastrointestinal Endoscopy*.

[12] ASGE Standards of Practice Committee, Anderson M. A., Fisher L. (2012). Complications of ERCP. *Gastrointestinal Endoscopy*.

[13] Dumonceau J.-M., Andriulli A., Elmunzer B. J. (2014). Prophylaxis of post-ERCP pancreatitis: European Society of Gastrointestinal Endoscopy (ESGE) guideline—updated June 2014. *Endoscopy*.

[14] Testoni P. A., Mariani A., Giussani A. (2010). Risk factors for post-ERCP pancreatitis in high-and low-volume centers and among expert and non-expert operators: a prospective multicenter study. *American Journal of Gastroenterology*.

[15] Nakai Y., Isayama H., Sasahira N. (2015). Risk factors for post-ERCP pancreatitis in wire-guided cannulation for therapeutic biliary ERCP. *Gastrointestinal Endoscopy*.

[16] Choudhary A., Bechtold M. L., Arif M. (2011). Pancreatic stents for prophylaxis against post-ERCP pancreatitis: a meta-analysis and systematic review. *Gastrointestinal Endoscopy*.

[17] Tham T. C. K., Kelly M. (2004). Association of periampullary duodenal diverticula with bile duct stones and with technical success of endoscopic retrograde cholangiopancreatography. *Endoscopy*.

[18] Poon R. T.-P., Yeung C., Liu C.-L. (2003). Intravenous bolus somatostatin after diagnostic cholangiopancreatography reduces the incidence of pancreatitis associated with therapeutic endoscopic retrograde cholangiopancreatography procedures: a randomised controlled trial. *Gut*.

[19] Rudin D., Kiss A., Wetz R. V., Sottile V. M. (2007). Somatostatin and gabexate for post-endoscopic retrograde cholangiopancreatography pancreatitis prevention: meta-analysis of randomized placebo-controlled trials. *Journal of Gastroenterology and Hepatology*.

[20] Bai Y., Xu C., Yang X., Gao J., Zou D.-W., Li Z.-S. (2009). Glyceryl trinitrate for prevention of pancreatitis after endoscopic retrograde cholangiopancreatography: a meta-analysis of randomized, double-blind, placebo-controlled trials. *Endoscopy*.

[21] Elmunzer B. J., Scheiman J. M., Lehman G. A. (2012). A randomized trial of rectal indomethacin to prevent post-ERCP pancreatitis. *The New England Journal of Medicine*.

[22] Kawaguchi Y., Ogawa M., Omata F., Ito H., Shimosegawa T., Mine T. (2012). Randomized controlled trial of pancreatic stenting to prevent pancreatitis after endoscopic retrograde cholangiopancreatography. *World Journal of Gastroenterology*.

[23] Qin Z., Linghu E.-Q. (2014). Temporary placement of a fully covered self-expandable metal stent in the pancreatic duct for aiding extraction of large pancreatic duct stones: preliminary data. *European Journal of Gastroenterology & Hepatology*.

[24] Kawahara I., Maeda K., Ono S. (2014). Surgical reconstruction and endoscopic pancreatic stent for traumatic pancreatic duct disruption. *Pediatric Surgery International*.

[25] Mazaki T., Mado K., Masuda H., Shiono M. (2014). Prophylactic pancreatic stent placement and post-ERCP pancreatitis: an updated meta-analysis. *Journal of Gastroenterology*.

[26] Glomsaker T., Søreide K., Hoff G., Aabakken L., Søreide J. A. (2011). Contemporary use of endoscopic retrograde cholangiopancreatography (ERCP): a Norwegian prospective, multicenter study. *Scandinavian Journal of Gastroenterology*.

[27] Cotton P. B., Garrow D. A., Gallagher J., Romagnuolo J. (2009). Risk factors for complications after ERCP: a multivariate analysis of 11,497 procedures over 12 years. *Gastrointestinal Endoscopy*.

[28] Cheng C.-L., Sherman S., Watkins J. L. (2006). Risk factors for post-ERCP pancreatitis: a prospective multicenter study. *The American Journal of Gastroenterology*.

[29] Mariani A., Giussani A., Di Leo M., Testoni S., Testoni P. A. (2012). Guidewire biliary cannulation does not reduce post-ERCP pancreatitis compared with the contrast injection technique in low-risk and high-risk patients. *Gastrointestinal Endoscopy*.

[30] Christoforidis E., Goulimaris I., Kanellos I., Tsalis K., Demetriades C., Betsis D. (2002). Post-ERCP Pancreatitis and hyperamylasemia: patient-related and operative risk factors. *Endoscopy*.

[31] Adbel Aziz A. M., Lehman G. A. (2007). Pancreatits after endoscopic retrograde cholangio-pancreatography. *World Journal of Gastroenterology*.

[32] Tammaro S., Caruso R., Pallone F., Monteleone G. (2012). Post-endoscopic retrograde cholangio-pancreatography pancreatitis: is time for a new preventive approach?. *World Journal of Gastroenterology*.

[33] Haciahmetoglu T., Ertekin C., Dolay K., Yanar F., Yanar H., Kapran Y. (2008). The effects of contrast agent and intraductal pressure changes on the development of pancreatitis in an ERCP model in rats. *Langenbeck's Archives of Surgery*.

[34] Sofuni A., Maguchi H., Mukai T. (2011). Endoscopic pancreatic duct stents reduce the incidence of post–endoscopic retrograde cholangiopancreatography pancreatitis in high-risk patients. *Clinical Gastroenterology and Hepatology*.

[35] Ito K., Fujita N., Noda Y. (2008). Pancreatic guidewire placement for achieving selective biliary cannulation during endoscopic retrograde cholangio-pancreatography. *World Journal of Gastroenterology*.

[36] Ito K., Fujita N., Kanno A. (2011). Risk factors for post-ERCP pancreatitis in high risk patients who have undergone prophylactic pancreatic duct stenting: a multicenter retrospective study. *Internal Medicine*.

[37] Ramesh J., Kim H., Reddy K., Varadarajulu S., Wilcox C. M. (2014). Impact of pancreatic stent caliber on post-endoscopic retrograde cholangiopancreatogram pancreatitis rates in patients with confirmed sphincter of Oddi dysfunction. *Journal of Gastroenterology and Hepatology*.

[38] Cot G. A., Kumar N., Ansstas M. (2010). Risk of post-ERCP pancreatitis with placement of self-expandable metallic stents. *Gastrointestinal Endoscopy*.

[39] Conigliaro R., Manta R., Bertani H. (2013). Pancreatic duct stenting for the duration of ERCP only does not prevent pancreatitis after accidental pancreatic duct cannulation: a prospective randomized trial. *Surgical Endoscopy and Other Interventional Techniques*.

[40] Frank C. D., Adler D. G. (2006). Post-ERCP pancreatitis and its prevention. *Nature Clinical Practice Gastroenterology & Hepatology*.

[41] Cheon Y. K., Cho K. B., Watkins J. L. (2007). Frequency and severity of post-ERCP pancreatitis correlated with extent of pancreatic ductal opacification. *Gastrointestinal Endoscopy*.

[42] Freeman M. L. (2007). Pancreatic stents for prevention of post-endoscopic retrograde cholangiopancreatography pancreatitis. *Clinical Gastroenterology and Hepatology*.

[43] Das A., Singh P., Sivak M. V., Chak A. (2007). Pancreatic-stent placement for prevention of post-ERCP pancreatitis: a cost-effectiveness analysis. *Gastrointestinal Endoscopy*.

